# Supplementary figures and images for: Reconstitution of an N-AChR from Brugia malayi, an evolved change in acetylcholine receptor accessory protein requirements in filarial parasites
Source: PLoS Pathog. 2022 Nov 14;18(11):e1010962. doi: 10.1371/journal.ppat.1010962 (PMC9714921; doi:10.1371/journal.ppat.1010962)

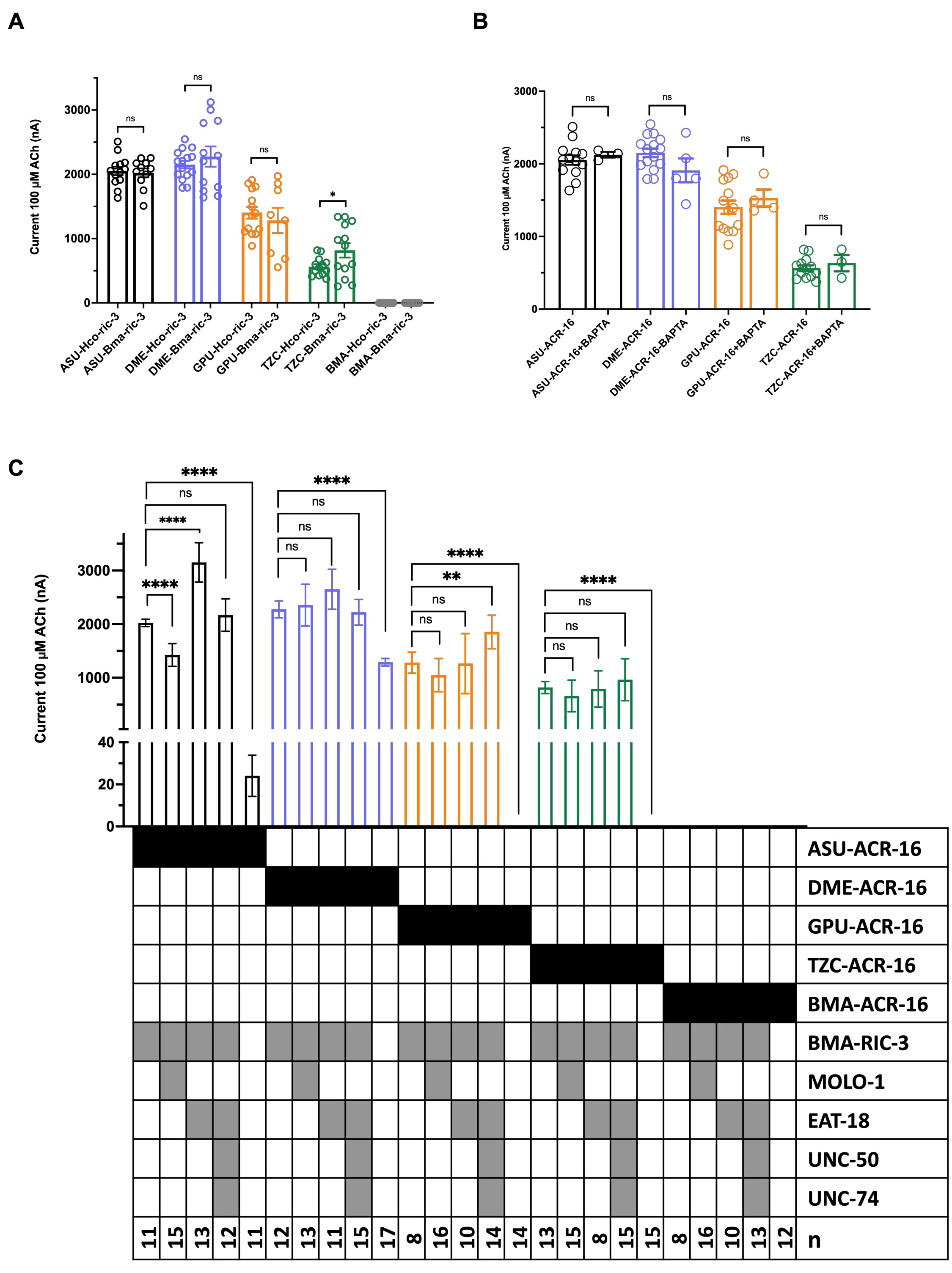

Supplement: S1 Fig — (A) Current responses of the clade III N-AChRs A. suum (black), D. medinensis (purple), G. pulchrum (orange), T. callipaeda (green) and B. malayi (grey) to 100 μM ACh when co-injected with either H. contortus RIC-3 (Hco-ric-3) or B. malayi RIC-3 (Bma-ric-3). A. suum ACR-16 with Hco-ric-3 (2052 ± 66 nA) was not different compared to co-injection with Bma-ric-3 (2023 ± 68 nA, p = 0.7581). D. medinensis ACR-16 with Hco-ric-3 (2150 ± 59 nA) was not different compared to co-injection with Bma-ric-3 (2275 ± 157 nA, p = 0.4285). G. pulchrum ACR-16 with Hco-ric-3 (1403 ± 91 nA) was not different compared to co-injection with Bma-ric-3 (1281 ± 197 nA, p = 0.5300). T. callipaeda ACR-16 with Hco-ric-3 (563 ± 39 nA) was smaller compared to co-injection with Bma-ric-3 (818 ± 112 nA, p<0.05). B. malayi ACR-16 produced no responses under both RIC-3 conditions. (B) Effect of BAPTA-AM incubation on current responses of the clade III N-AChRs. A. suum (black), D. medinensis (purple), G. pulchrum (orange) and T. callipaeda (green) responses to 100 μM ACh co-injected with H. contortus RIC-3. A. suum ACR-16 (2052 ± 70 nA) was not different compared to BAPTA-AM incubated ACR-16 (2125 ± 40 nA, p = 0.6193). D. medinensis ACR-16 (2150 ± 59 nA) was not different compared to BAPTA-AM incubated ACR-16 (1910 ± 164 nA, n = 0.0969). G. pulchrum ACR-16 (1403 ± 91 nA) was not different compared to BAPTA-AM incubated ACR-16 (1529 ± 117 nA, n = 0.5044). T. callipaeda ACR-16 (563 ± 39 nA) was not different compared to BAPTA-AM incubated ACR-16 (623 ± 113 nA, n = 0.4829). Note that the BAPTA-AM treated oocytes represents preliminary data. (C) Current responses of clade III N-AChRs A. suum (black), D. medinensis (purple), G. pulchrum (orange), T. callipaeda (green) and B. malayi (grey) to 100 μM ACh with different accessory proteins. Receptor responses are compared to Bma-RIC-3 condition. Error bars represent standard error. n>8, *p<0.05; **p<0.01; ****p<0.0001. See S1 Table for values. (TIFF) [file ppat.1010962.s001.tiff]

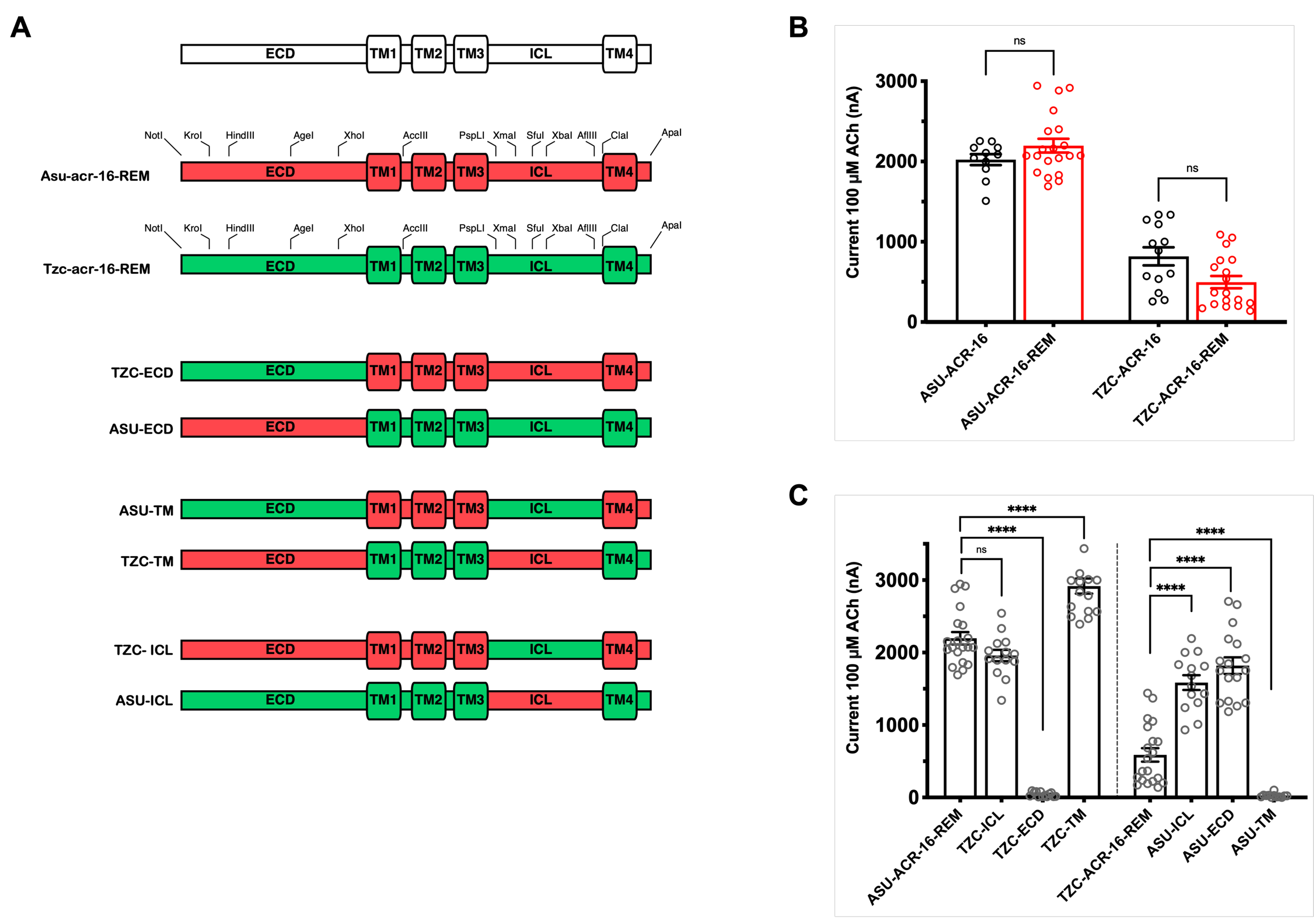

Supplement: S2 Fig — The three main structural regions (ECD, ICL & TM) were exchanged between A. suum and T. callipaeda ACR-16 to identify the regions mediating the declining responses in later clade III N-AChRs. (A) A. suum and T. callipaeda ACR-16 coding sequences were modified to introduce restriction enzyme sites to constrict chimeras. A. suum shown in red and T. callipaeda in green. The modified sequences introduced complementary restriction enzyme sites termed ACR-16-REM. The three main structural regions (ECD, ICL, TMs) were exchanged between the two receptors. TZC-ECD contains the A. suum sequence with the T. callipaeda ECD. ASU-ECD contains the T. callipaeda sequence with the A. suum ECD. ASU-TM contains the T. callipaeda sequence with the A. suum TM. TZC-TM contains the A. suum sequence with the T. callipaeda TM. TZC-ICL contains the A. suum sequence with the T. callipaeda ICL. ASU-ICL contains the T. callipaeda sequence with the A. suum ICL. (B) Coding-sequence modified ACR-16 receptors (ACR-16-REM, red) produced responses indistinguishable from native ACR-16 sequence (black). Asu-ACR-16 (2023 ± 68 nA, n = 11) versus Asu-ACR-16-REM (2197 ± 383 nA, n = 20); and Tzc-ACR-16 (818 ± 112 nA, n = 13) versus Tzc-ACR-16-REM (587 ± 415 nA, n = 20). B. malayi ric-3 co-injected with all. Circles represent currents measured from individual oocytes. Error bars represents standard error. (C) Chimeras exchanging each of the three structural regions between the receptors identified all regions contribute to the declining current responses measured in later clade III receptors. Circles represent currents measured from individual oocytes. Error bars represents standard error. Significance determined by comparing the current responses to the relevant reference receptor. ****p<0.0001. (TIFF) [file ppat.1010962.s002.tiff]

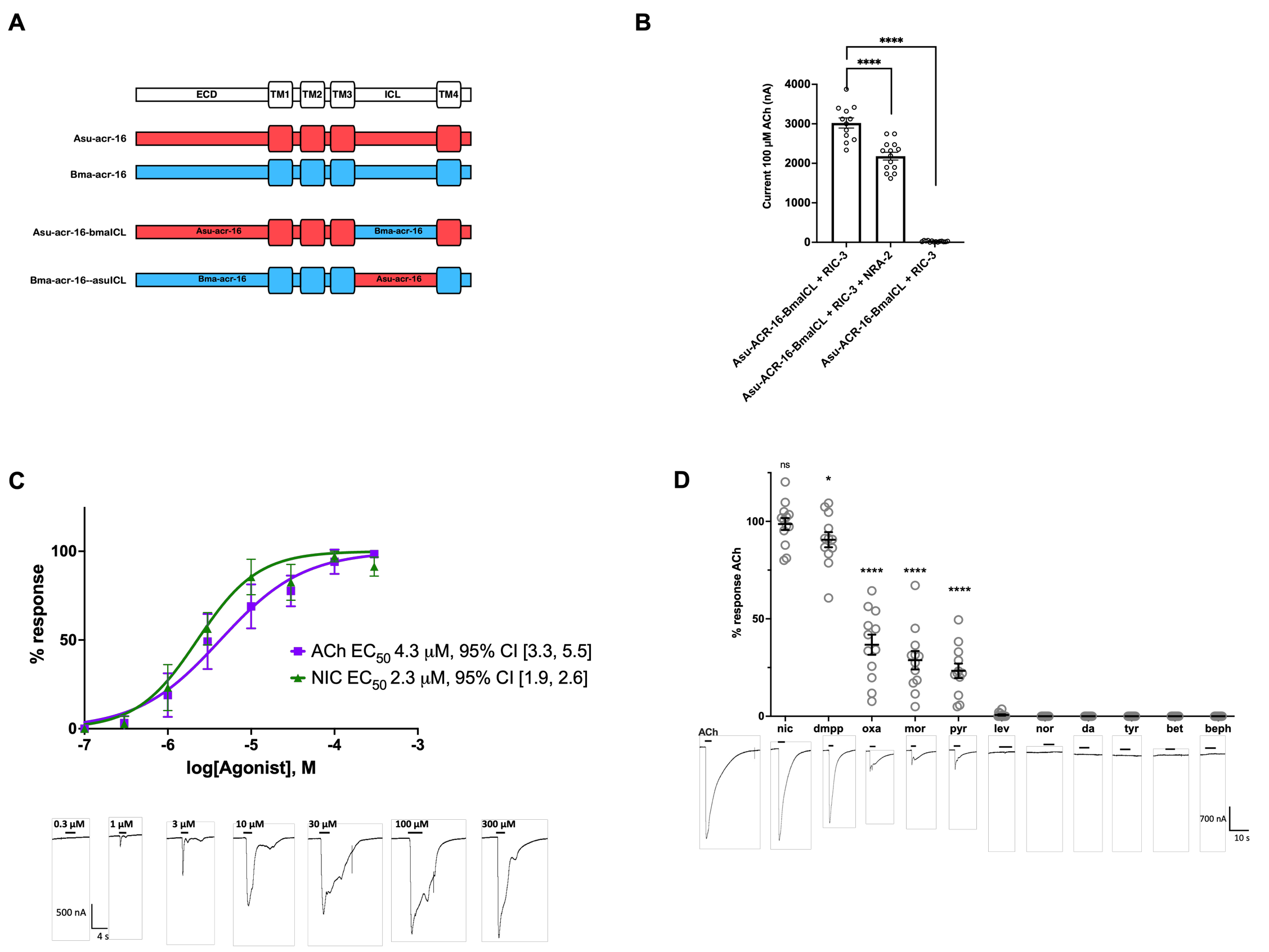

Supplement: S3 Fig — (A) Chimeras for the A. suum (red) and B. malayi acr-16 (blue) sequences. Asu-ACR-16-bmaICL chimera has the Asu-acr-16 subunit with the intracellular loop of Bma-acr-16, and Bma-ACR-16-asuICL chimera has the Bma-acr-16 subunit with the intracellular loop of Asu-acr-16. Chimeras were made between A. suum and B. malayi ACR-16 to characterize B. malayi ACR-16 in the event that the unmodified B. malayi ACR-16 did not produce robust enough responses for characterization. The intracellular loop was exchanged since it is one of the regions thought to contribute to the declining responses observed in clade III ACR-16 and it does not contain the ligand-binding regions. (B) Asu-ACR-16-bmaICL chimera current responses under different accessory protein conditions: RIC-3 (3021 ± 444 nA, n = 12); RIC-3 + NRA-2 (2181 ± 369 nA, n = 14, p<0.0001); and no accessory protein (22 ± 15 nA, n = 14, p<0.0001). Circles represent current responses from individual oocytes. Error bars represent standard error. ****p<0.0001. (C) Concentration-response curves were measured for the Asu-ACR-16-bmaICL chimera to compare to the Asu-ACR-16 receptor as a control (see Fig 4A and 4B). Concentration-response curves for acetylcholine in purple (EC50 = 4.3 μM, 95% CI [3.3, 5.5], n = 8) and nicotine in green (EC50 = 2.3 μM, 95% CI [1.9, 2.6], n = 10). Hill coefficients were 0.9, 95% CI [0.7, 1.1] for ACh and 1.2, 95% CI [0.9, 1.6] for NIC. Sample recordings to acetylcholine is shown. Error bars represent standard error. (D) Asu-ACR-16-bmaICL chimera response to ligands relative to acetylcholine. Concentrations of 100 μM was used for all ligands. Apart from higher responses to DMPP, Asu-ACR-16-bmaICL chimera had a comparable response profile compared to Asu-ACR-16 (see Fig 5A): ACh = DMPP> NIC> OXA = MOR = PYR > LEV = EPI = NOR = DA = TYR = BET = BEPH. Sample recording to each ligand is shown. Error bars represent standard error. *p<0.05; ****p<0.0001. (TIFF) [file ppat.1010962.s003.tiff]

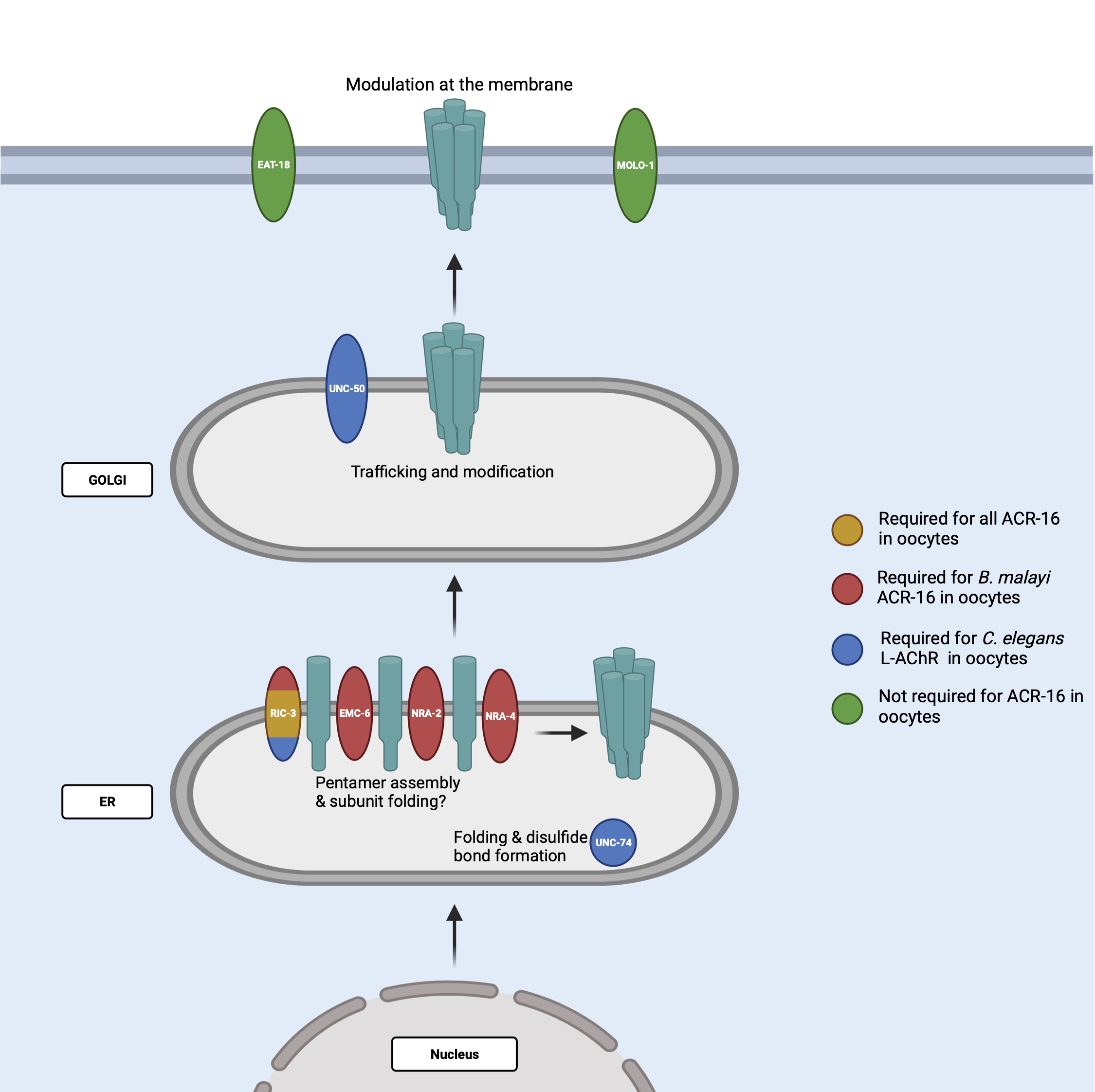

Supplement: S4 Fig — Expected effect on receptor synthesis of accessory proteins included in the present study. Ric-3 (yellow, blue, red) is required for all nematode ACR-16 and the L-AChR in oocytes and is involved subunit assembly in the ER. EMC-6, NRA-2 and NRA-4 (red) are required for the B. malayi ACR-16 receptor in oocytes and are thought to be involved in subunit folding and oligomerization of the pentamer in the ER. UNC-74 (blue) is required for disulfide bond formation in the ER for the L-AChR. UNC-50 (blue) is involved in L-AChR receptor trafficking through the Golgi. EAT-18 (green) is required for the EAT-2 receptor at the membrane and MOLO-1 (green) is required for the L-AChR in vivo and modulates receptor response. See text for detailed discussion. (TIFF) [file ppat.1010962.s004.tiff]
