# Supplementary material for: Reconstitution of an N-AChR from Brugia malayi, an evolved change in acetylcholine receptor accessory protein requirements in filarial parasites
Source: PLoS Pathog. 2022 Nov 14;18(11):e1010962. doi: 10.1371/journal.ppat.1010962 (PMC9714921; doi:10.1371/journal.ppat.1010962)
Supplement: S1 Table — Clade III N-AChR responses (nA) to 100 μM acetylcholine under various accessory protein combinations. The receptor responses are compared to the reference condition of the N-AChR + B. malayi RIC-3. The clade III receptors showed declining currents along their phylogeny with the RIC-3 accessory protein; A. suum and D. medinensis N-AChR produced the largest response currents, G. pulchrum N-AChR produced smaller responses, T. callipaeda N-AChR had even smaller responses, and the B. malayi N-AChR had none. This decline was seen even with the addition of accessory proteins EAT-18, UNC-50, UNC-74 and/or MOLO-1. A B. malayi N-AChR response was only measured upon addition of EMC-6, NRA-2 or NRA-4, however despite multiple combinations of these three accessory proteins, small responses were still obtained indicating that other critical accessory proteins are still missing from the injection mixture. Chimera Bma-ACR-16-asuICL was used to approximate B. malayi N-AChR receptor pharmacology. Error represents standard error. *p<0.05; **p<0.01; ***p<0.0005; ****p<0.0001. (DOCX) [file ppat.1010962.s005.docx]

|  | **Asu-ACR-16** | **Dme-ACR-16** | **Gpu-ACR-16** | **Tzc-ACR-16** | **Bma-ACR-16** | **Bma-ACR-16-asuICL** | **Asu-ACR-16-bmaICL** |
| --- | --- | --- | --- | --- | --- | --- | --- |
| **Bma-ric-3 Reference condition** | 2023 ± 68  (n=11) | 2275 ± 157  (n=12) | 1281 ± 197  (n=8) | 818 ± 112  (n=13) | 0 ± 0  (n=8) | 59 ± 12 (n=14) | 3021 ± 128 (n=12) |
| **Hco-ric-3** | 2052 ± 66  (n=13) ns | 2150 ± 59  (n=15) ns | 1403 ± 91  (n=14)  ns | 563 ± 39  (n=13)  * | 0 ± 0  (n=12) | - | - |
| **Bma-ric-3**  **Bma-unc-50**  **Bma-unc-74** | 2606 ± 183  (n=10) ** | 2368 ± 105  (n=13)  ns | 847 ± 124  (n=14)  ns | 367 ± 43  (n=15)  *** | 0 ± 0  (n=5) | - | - |
| **Bma-ric-3**  **Bma-molo-1** | 1425 ± 55  (n=15)  **** | 2354 ± 108  (n=13)  ns | 1049 ± 77  (n=16)  ns | 660 ± 76  (n=15)  ns | 0 ± 0  (n=16) | - | - |
| **Bma-ric-3**  **Bma-eat-18** | 3152 ± 102  (n=13) **** | 2649 ± 112  (n=11)  ns | 1264 ± 177  (n=10)  ns | 791 ± 119  (n=8)  ns | 0± 0  (n=10) | - | - |
| **Bma-ric-3 Bma-unc-50**  **Bma-unc-74**  **Bma-eat-18** | 2166 ± 87  (n=12) ns | 2222 ± 62  (n=15)  ns | 1854 ± 83  (n=14)  ** | 964 ± 101  (n=15)  ns | 0 ± 0  (n=13) | - | - |
| **Bma-ric-3**  **Cel-emc-6** | 2582 ± 89 (n=12) **** | 2183 ± 87 (n=11)  ns | 1628 ± 123 (n=17)  ns | 720 ± 70 (n=13)  ns | 17 ± 4 (n=13)  ** | 37 ± 12 (n=15)  ns | - |
| **Bma-ric-3**  **Cel-nra-2**  **Cel-nra-4** | 2849 ± 152 (n=14) *** | 2425 ± 133 (n=12)  ns | 2553 ±123 (n=12)  **** | 1263 ± 88 (n=15)  ** | 4 ± 2 (n=12)  * | 47 ± 18 (n=14)  ns | - |
| **Bma-ric-3**  **Cel-nra-2** | - | - | - | - | 33 ± 9 (n=14)  * | 256 ± 89 (n=11)  * | 2181 ± 99 (n=14)  **** |
| **Bma-ric-3**  **Cel-nra-4** | - | - | - | - | 31 ± 13 (n=12)  ns | - | - |
| **Bma-ric-3**  **Bma-emc-6**  **Cel-nra-2**  **Cel-nra-4** | - | - | - | - | 11 ± 9 (n=11)  ns | 1 ± 1 (n=11)  *** | - |
| **Bma-ric-3**  **Bma-emc-6**  **Cel-nra-2**  **Cel-nra-4**  **Bma-eat-18** | - | - | - | - | 2 ± 1 (n=12)  ns | - | - |
| **Bma-ric-3**  **Bma-emc-6**  **Cel-nra-2**  **Cel-nra-4**  **Bma-eat-18**  **Bma-unc-50**  **Bma-unc-74** | - | - | - | - | 0 ± 0 (n=11) | - | - |
| **No accessory protein** | 24 ± 10  (n=11) **** | 1290 ± 70  (n=17)  **** | 0 ± 0  (n=14)  **** | 0 ± 0  (n=15)  **** | 0 ± 0  (n=12) | 0 ± 0 (n=15)  **** | 22 ± 4 (n=14)  **** |
