# Supplementary material for: Reconstitution of an N-AChR from Brugia malayi, an evolved change in acetylcholine receptor accessory protein requirements in filarial parasites
Source: PLoS Pathog. 2022 Nov 14;18(11):e1010962. doi: 10.1371/journal.ppat.1010962 (PMC9714921; doi:10.1371/journal.ppat.1010962)
Supplement: S2 Table — Receptor subunit and accessory protein coding sequences used in this study were either cloned from worm cDNA or synthesized based on their genome-predicted sequences. Bma-acr-16 and B. malayi accessory proteins were cloned from adult female cDNA using the following primers. All other accessory proteins and subunit sequences were synthesized. Experimental procedure is described in Methods. a received from Dr. C. Neveu and Dr. C. Charvet. (DOCX) [file ppat.1010962.s006.docx]

**S2 Table. Primers used for cloning.**

| **Gene** | **Cloned** | **Synthesized** | **Outer Forward primer (5’-‘3)** | **Outer Reverse primer (5’-‘3)** | **Inner Forward primer (5’-‘3)** | **Inner Reverse primer (5’-‘3)** |
| --- | --- | --- | --- | --- | --- | --- |
| ***Asu-acr-16*** |  | Yes |  |  |  |  |
| ***Dme-acr-16*** |  | Yes |  |  |  |  |
| ***Gpu-acr-16*** |  | Yes |  |  |  |  |
| ***Tzc-acr-16*** |  | Yes |  |  |  |  |
| ***Bma-acr-16*** | Yes |  | AAAAACGAAAGAAGGAAATTCCAAAA | TTATAATAATAATGAAATTAAATTAAGC | TGAATATATGCCAAGCGGTGAATG | CTTCTGAGGTTGGTGGTGACAT |
| ***Asu-acr-16-REM*** |  | Yes |  |  |  |  |
| ***Tzc-acr-16-REM*** |  | Yes |  |  |  |  |
| ***Asu-acr-16-bmaICL*** |  | Yes |  |  |  |  |
| ***Bma-acr-16-asuICL*** |  | Yes |  |  |  |  |
| ***Hco-ric-3*** | Yes ^a^ |  |  |  |  |  |
| ***Bma-ric-3*** | Yes |  | GAAGGCTTGCTCTCTCTTGGT | TTGGGTCCATAAGTTGTTGATGT | GCGGCCGCCGTTGAATGTCAGCGGAACC | GGGCCCTTAGATTCTTAACTTTTTCCACG |
| ***Bma-unc-50*** | Yes |  | CTGACGATTGATCAGCTACTGC | TCAGAAGCATATGCATATCACT | GCGGCCGCCTACTGCAGCTGTTTCACA | GGGCCCGCAAATATTCTATTCTCAACTTCCTCT |
| ***Bma-unc-74*** | Yes |  | GCAAGGATTTTGAATATCCGTAATTT | ACGCTTCTGCTGGATTTCATC | GCGGCCGCTGTGAATGTGGTTATTTAATTGTTGC | GGGCCCCACATCCAACTTTTACTCCTCCT |
| ***Bma-molo-1*** | Yes |  | CTCGTTAATAATAATTCAAATAATGGC | ACAATAATCAATTTATGAAATTATACA | GCGGCCGCTGTAAAATGAGCCTAACACATTGG | GGGCCCTTTTTGAATCAAACAAATGTTGCAT |
| ***Bma-eat-18*** |  | Yes |  |  |  |  |
| ***Cel-emc-6*** |  | Yes |  |  |  |  |
| ***Cel-nra-2*** |  | Yes |  |  |  |  |
| ***Cel-nra-4*** |  | Yes |  |  |  |  |
